# The ISGylation tapestry in cancer: weaving phenotypic plasticity through multidimensional regulatory looms

**DOI:** 10.1186/s11658-025-00815-6

**Published:** 2025-11-05

**Authors:** Ruicheng Wu, Fanglin Shao, Siang Boon Koh, Uzoamaka Adaobi Okoli, Dengxiong Li, Jie Wang, Zhouting Tuo, Rong Zhang, Dilinaer Wusiman, Umber Cheema, Depei Kong, Dechao Feng

**Affiliations:** 1 Urology & Nephrology Center, Department of Urology, Affiliated People’s Hospital, Zhejiang Provincial People’s Hospital, Hangzhou Medical College, Hangzhou, Zhejiang China; 2 Department of Urology, Institute of Urology, West China Hospital, Sichuan University, Chengdu, 610041 China; 3 Division of Surgery & Interventional Science, University College London, London, W1W 7TS UK; 4 Department of Rehabilitation, The Affiliated Hospital of Southwest Medical University, Luzhou, 646000 People’s Republic of China; 5 Chengdu Basebio Company, Chengdu, China; 6 Faculty of Health and Life Sciences, University of Bristol, Bristol, BS8 1TD UK; 7 Basic and Translational Cancer Research Group, Department of Pharmacology and Therapeutics, College of Medicine, University of Nigeria, Nsukka, Enugu Nigeria; 8 Department of Urological Surgery, Daping Hospital, Army Medical Center of PLA, Army Medical University, Chongqing, China; 9 Clinical Medicine Research Institute, Zhejiang Provincial People’s Hospital of Hangzhou Medical College, Hangzhou, China; 10 Department of Comparative Pathobiology, College of Veterinary Medicine, Purdue University, West Lafayette, IN 47907 USA; 11 Purdue Institute for Cancer Research, Purdue University, West Lafayette, IN 47907 USA

**Keywords:** Post-translational modification, ISGylation, Tumor microenvironment, Immune evasion, Drug resistance

## Abstract

Post-translational modification is an important mechanism for regulating protein function and cell signaling networks. Among these modifications, ISGylation is a ubiquitin-like modification regulated by ISG15. In this review, we explore the role of ISGylation in a variety of related phenotypes in the tumor context, including apoptosis regulation, autophagy regulation, immune escape, metabolic reprogramming, cancer stem cell maintenance, and DNA damage repair. ISGylation plays a dual role in apoptosis, promoting either pro-survival or pro-death pathways depending on contexts. It also regulates autophagy by promoting tumor adaptation or by regulating immune responses. Moreover, ISGylation contributes to the immune escape mechanism by regulating the stability of PD-L1 and immune cell infiltration. In addition, ISGylation is involved in metabolic reprogramming, supporting tumor growth and therapeutic resistance by regulating key metabolic pathways. It also plays a key role in maintaining the properties of cancer stem cells by stabilizing essential metabolic and signaling proteins. In sum, this review examines the functions and mechanisms of ISG15 and ISGylation in various tumor-associated phenotypes, enhancing our understanding of their role in tumorigenesis and disease progression.

**Graphical abstract:**

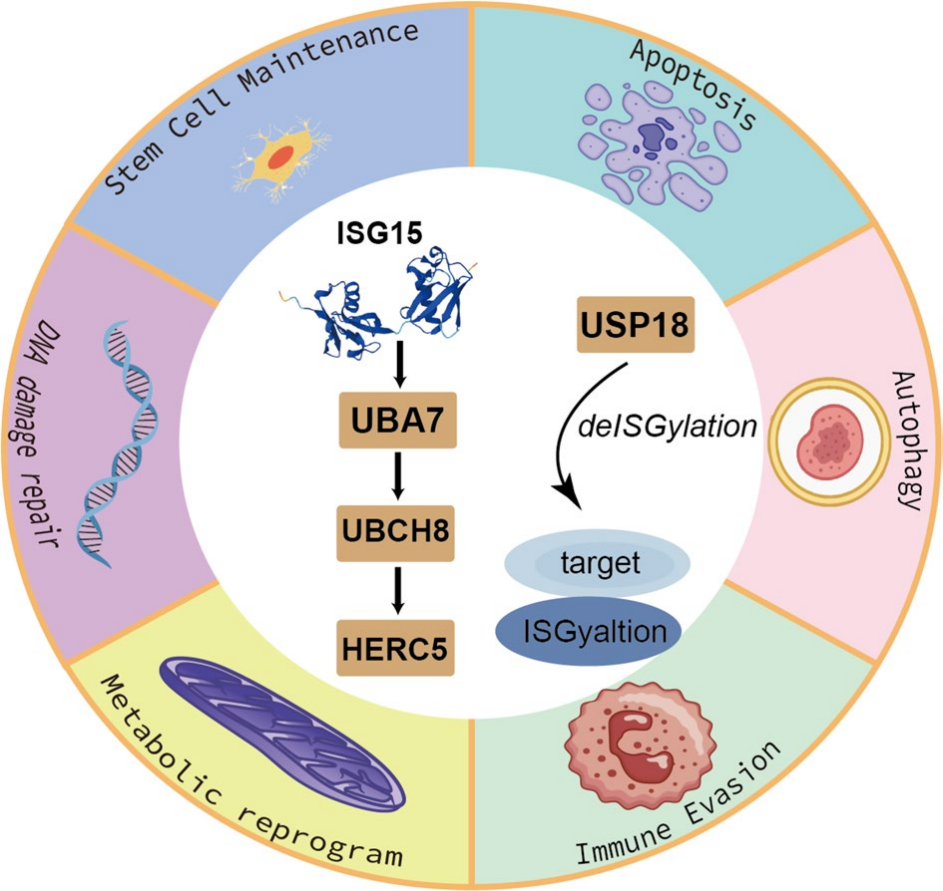

**Supplementary Information:**

The online version contains supplementary material available at 10.1186/s11658-025-00815-6.

## Introduction

Post-translational modification (PTM) is an important mechanism for regulating protein function and cell signaling network dynamics [1]. It occurs following protein synthesis and significantly affects the function of proteins by altering their structure, activity, localization, and stability, thereby reshaping cellular processes and contributing to the progression of diseases [2–4]. ISGylation is a unique ubiquitin-like modification that regulates protein function through covalent binding of interferon-stimulated gene 15 (ISG15) to target proteins [5]. The level of modification is regulated by type I interferon (IFN-I) signaling, and involves a series of enzymatic reactions mediated by specific E1 (activating enzyme), E2 (conjugating enzyme), and E3 (ligase) [6, 7]. ISG15 and enzymes that participate in the ISGylation cascade—UBA7, UBCH8, and HERC5—are themselves interferon-stimulated genes (ISGs) that are transcriptionally induced by IFN-I [8]. The process of ISGylation consists of three main steps. Firstly, ISG15 is activated in an adenosine triphosphate (ATP)-dependent manner through E1 activating enzyme UBA7; subsequently, the E2 conjugating enzyme UBCH8 accepts the activated ISG15 and transfers it to the target protein; finally, E3 ligase (HERC5) catalyzes the covalently binding of ISG15 to lysine residues of the substrate protein [6, 9]. De-ISGylation is mediated by de-ISGylation enzymes (USP18) that remove ISG15 modifications from target proteins, thereby achieving dynamic regulation [10, 11]. The main difference between ISGylation and other similar ubiquitin-like modifications is that ubiquitin is mainly associated with the proteasome degradation pathway, while ISGylation has a unique substrate specificity and does not target protein degradation but regulates protein function [12, 13]. In addition, ISG15 can conjugate to ubiquitin at Lys29 to form ISG15–ubiquitin mixed chains. These atypical chains were shown to impair proteasomal degradation of ubiquitylated proteins in cell-based reporter assays and in vitro conjugation systems [14]. However, this mechanism needs to be further verified. Independently of ISGylation changes induced on targets, free ISG15 has important functions inside and outside the cell. Intracellular free ISG15 inhibits the excessive enhancement of IFN-I responses by stabilizing USP18, thereby preventing autoinflammatory responses [15]. Extracellular ISG15 functions as a cytokine-like molecule to activate immune cells and stimulate secretion of cytokines by binding to surface receptors on cells [16, 17]. The conjugation-independent functions of ISG15 are essential for its immune regulation function.

As an immune-related ubiquitin-like modifier, ISG15 exerts its biological functions through both conjugation (ISGylation) and unconjugated (free) forms. Initially identified for its role in antiviral immunity, ISG15 was first studied in the field of antiviral immunity and was shown to play an important role in interfering with the replication and assembly of viral proteins and enhancing the ability of cells to resist pathogens [18, 19]. In recent years, increasing number of studies have shown that ISG15 has a complex regulatory role in the tumor microenvironment (TME) and tumor progression, and may have different effects in different cancers [20, 21]. Depending on the cancer type, cellular context, and immune status, ISG15 may act either as a tumor suppressor or as a promoter of malignancy. In this review, we will summarize the novel roles of ISG15 in multiple cancer-related phenotypes, including apoptosis, autophagy, immune escape, metabolic reprogramming, cancer stem cells (CSCs) maintenance, and DNA damage response (DDR) resistance. Finally, we aim to highlight future research directions to better elucidate the multifaceted role of ISG15 in tumors. The graphical abstract (Supplementary Fig. 1) provides an overview of the main concepts and structure of this review. All graphics were created with Figdraw(www.figdraw.com) and Adobe Illustrator (Adobe Systems, San Jose, CA, USA).

## ISGylation and tumor apoptosis

The function of ISG15 expression in the apoptotic phenotype of tumor cells is dependent on multiple factors. Lim et al. [22] have found that ISG15 covalently modifies CTBP1 under the stimulation of IFN-I, which in turn enhances the formation of a transcriptional repressor complex involving HDAC1 and LSD1. ISGylation enhances CTBP1-mediated repression of BAX and E-cadherin. Studies have also shown that ISG15 is regulated by microRNAs. For example, miR-370 downregulates ISG15 expression by targeting its 3’UTR, thereby enhancing the anti-tumor activity of IFN-I in hepatocellular carcinoma (HCC) cells [23]. While ISGylation is not commonly considered a classical degradation signal, the UBA7-mediated ISGylation of cyclin D1 can destabilize the protein and impede the growth of lung cancer cells [24]. USP18 can remove ISGylation and stabilize cyclin D1, thereby promoting cell proliferation and inhibiting cell apoptosis. It can also increase the cell response to cisplatin and be used as a potential intervention target [25]. In addition to the traditional ISGylation-dependent pathway, free ISG15 has intracellular pro-apoptotic activity. Mao et al. [20] showed that ISG15 expression induced by anticancer drugs such as clioquinol and mefloquine can downregulate key NF-κB components such as IKKβ and p65 and reduce their phosphorylation levels, thus promoting tumor cell apoptosis. Similarly, Wan et al. [26] reported that treatment with IFN-I induced expression of ISG15 in HepG2 cells but did not induce apoptosis. In contrast, overexpression of ISG15 significantly promoted apoptosis and cell cycle arrest. These results suggest that endogenous ISG15 induced by IFN-I signaling may be functionally inhibited, whereas exogenous overexpression of ISG15 may have tumor suppressive activity. Interestingly, IFN-I can upregulate the expression of TRAIL, BAX, and other pro-apoptotic genes by activating STAT1/2, and can also upregulate the expression of ISG15 [27]. The reason may be that ISGylation inhibits the transcription of pro-apoptotic genes on the one hand, and inhibits ISGs by maintaining USP18 stability on the other hand [28].

**Fig. 1:**
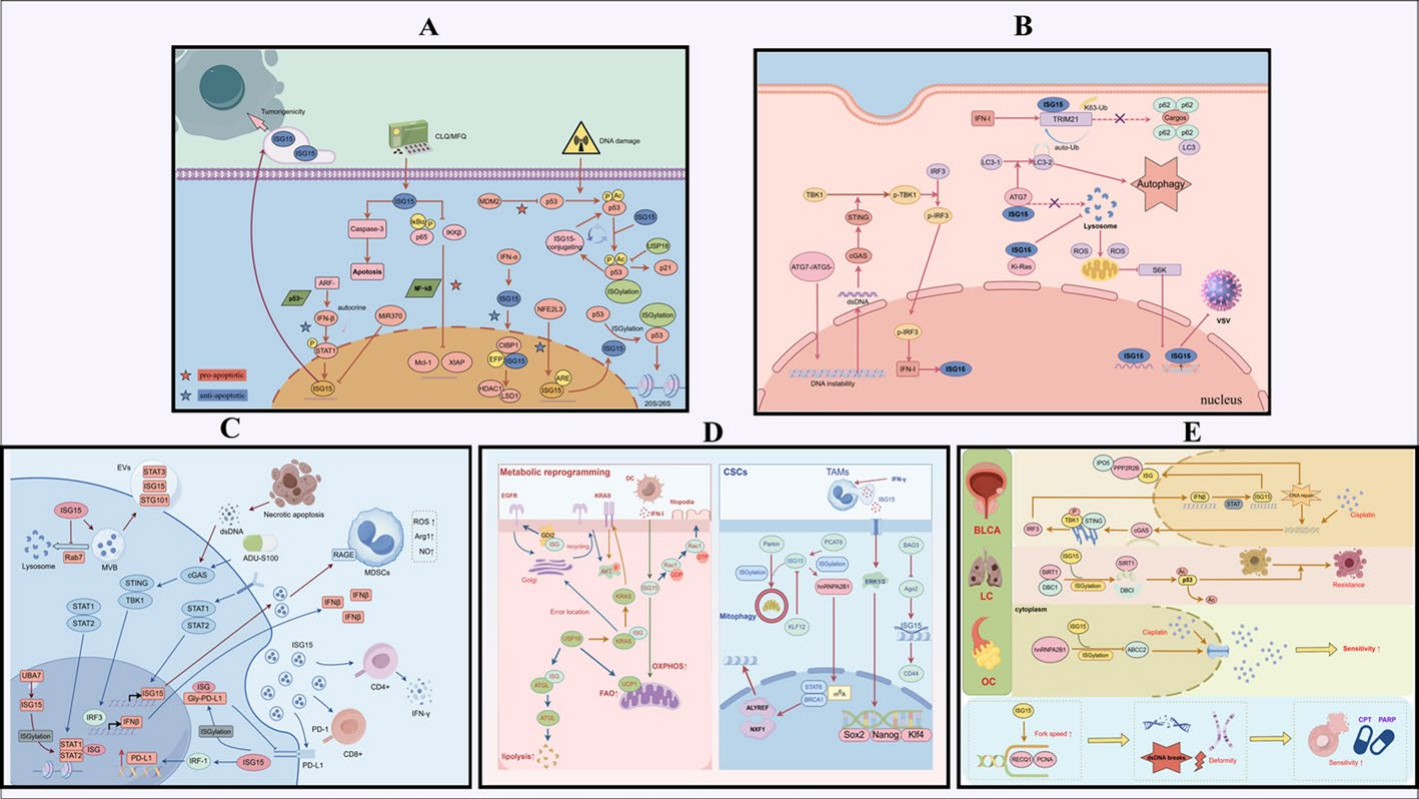
The regulatory network of ISG15 and ISGylation in cancer biology.**A** ISG15 and ISGylation modulate apoptotic signaling in cancer cells. **B** ISG15 and ISGylation are involved in autophagy-related pathways. **C** ISG15 and ISGylation contribute to immune escape mechanisms. **D** ISGylation shapes cellular metabolism and sustains CSC traits. **E** ISG15 and ISGylation influence DNA repair machinery and chemoresistance. BLCA, bladder cancer; cGAS, cyclic GMP–AMP synthase; CLQ, chloroquine; CPT, camptothecin; CSC, cancer stem cell; DC, dendritic cell; dsDNA, double-stranded DNA; EGFR, epidermal growth factor receptor; EVs, extracellular vesicles; IFN-α, interferon-alpha; IFN-β, interferon-beta; IFN-γ, interferon-gamma; IFN-I, type I interferon; LC, lung cancer; MFQ, mefloquine; NO, nitric oxide; OC, ovarian cancer; PARP, poly(ADP-ribose) polymerase; PCNA, proliferating cell nuclear antigen; PD-L1, programmed death-ligand 1; ROS, reactive oxygen species; STING, stimulator of interferon genes; VSV, vesicular stomatitis virus. All graphics were created with Figdraw (www.figdraw.com) and Adobe Illustrator (Adobe Systems, San Jose, CA, USA).

As a major regulator, p53 can activate a series of pro-apoptotic genes to induce programmed cell death [29, 30]. Recent studies have found that there is a complex regulatory network between ISG15 and p53. Further, ISGylation can promote p53 degradation and inhibit its pro-apoptotic function. Huang et al. [31] found that HERC5-mediated ISGylation can accelerate the degradation of p53 by proteasome. Ren et al. [32] showed that NFE2L3 directly induces ISG15 expression and promotes p53 degradation by enhancing ISGylation of p53, thereby inhibiting the apoptosis of HCC cells. The weaker effect of this mechanism in mice suggests possible species-specific differences. In addition, oncogenes such as Src, Ras, and Myc can also enhance the binding of p53 to HERC5 by promoting the phosphorylation of Tyr126/220, promoting the degradation of p53 and weakening the anti-apoptosis ability of tumor cells [33]. Park et al. [34] revealed a protective role of p53 ISGylation under DNA damage conditions. p53 induces the expression of ISG15 binding system (UBE1L, UBCH8, TRIM25), which leads to the ISGylation of p53 at Lys291/292. Enhanced p53 phosphorylation, acetylation, and DNA binding affinity form a positive feedback loop that drives transcription of pro-apoptotic genes such as BAX. However, in the absence of p53, free ISG15 may promote tumor progression. ARF is a tumor suppressor involved in the regulation of p53-independent stress responses. Forys et al. [35] found that ISG15 was upregulated by the IFN-I-STAT1 axis in p53-null cells, and ARF deletion resulted in continuous activation of this pathway; ISG15 thereby mediates tumor-promoting effects through both intracellular and extracellular mechanisms. We hypothesize that the tumor-suppressive effect of free ISG15 might be related to the expression of p53, and that free ISG15 acts as a tumor promoter when p53 expression is absent. These functional differences may reflect the role of ISG15 in specific contexts, depending on cell type differences, p53 status, and induction of upstream molecules. The above relevant core literature information is presented in Table 1, and Fig. 1A shows the proposed mechanism.

**Table 1:** Key references categorized by functional phenotype related to ISGylation

Type	PMID	Year	Cancer type	Vitro model	Vivo model	Key findings
Apoptosis	27,659,523	2016	AML, CC and MM	K562, OCI-AML2, RPMI-8226, HeLa, and HEK293T	BALB/c female nude mice	ISG15 promotes caspase-3-dependent apoptosis by decreasing the expression of key NF-κB-related proteins
	38,405,356	2024	CC	HeLa, HEK293T, and MEFs	NA	ISG15 enhances the inhibition of EMT and apoptosis-related genes by modulating the interaction of CtBP1 and its binding partners
	29,758,929	2018	HCC	HLCZ01, LH86, SMMC7721, Huh7, and LO2	Male NOD/SCID mice	miR-370 downregulates the expression of ISG15 mRNA 3'-UTR by targeting it, thereby enhancing IFN-α-induced apoptosis of HCC cells
	22,752,428	2012	LC	A549, H23, HOP62, ED-1, PT67, and 293	Female FVB syngeneic mice	USP18 inhibits lung cancer cell apoptosis by stabilizing Cyclin D1 and reduces its sensitivity to cisplatin
	24,844,324	2014	CRC and BRCA	HCT116, MCF7, HEK293T, and MEFs	ISG15-null mice	ISGylation promotes p53 degradation through HERC5-mediated modification and primarily acts on misfolded p53
	25,071,020	2014	CRC and BRCA	HCT116, MCF7, and HEK293T	ISG15-null and p53-null mice	Src-mediated phosphorylation of p53 Tyr126 and Tyr220 enhances the ISGylation of p53, thereby accelerating the degradation of p53 and reducing its tumor inhibitory activity
	24,726,362	2014	BRCA	Primary embryonic fibroblasts	FVB.129 mice	ARF limits the proliferation and tumor formation of p53-deficient cells by inhibiting the IFN-β-STAT1-ISG15 axis
	37,350,063	2023	HCC	HepG2 and MHCC97H	Male nude mice	NFE2L3 activates ISG15 through transcription, promotes the ISGylation of p53, and accelerates the degradation of its 26S proteasome, thus promoting the proliferation of HCC cells
	24,024,201	2013	HCC	HepG2	NA	Overexpression of ISG15 can enhance the expression of p53 and p21, promote protein ubiquitination, inhibit the proliferation of IFN-α-resistant HCC cells, and induce apoptosis
	27,545,325	2016	Multitumor	HCT116, HEK293T, H1299, and A549	BALB/c male nude mice	ISG15-mediated p53 ISGylation forms a positive feedback loop that enhances p53 transcriptional activity and inhibiting tumor growth
Autophagy	34,257,278	2021	NSCLC	A549 and HEK293	NA	ISGylation regulates autophagy through p62 ubiquitination mediated by TRIM21
	32,127,658	2020	HCC, BRCA and FS	HT1080, MCF7, HepG2, MEFs, and NIH3T3	NA	Loss of ATG5 and ATG7 activates tumor-associated phenotypes through STING-induced ISG15 expression
	38,385,083	2024	PDAC	PANC-1 and MIA PaCa-2	Balb/c mice	ISG15 promotes autophagy by binding to and stabilizing ATG7, thereby enhancing tumor cell survival and tolerance to gemcitabine
	23,318,454	2014	BRCA	MDA-MB-231, MCF-10A, and NIH3T3	NA	By inhibiting the lysosomal degradation of Ki-Ras and stabilizing its protein expression, ISG15 promotes the proliferation, migration, and invasion of tumor cells
	28,186,990	2017	ECA	OE21, OE33, KYSE450, and OE19	NA	Knocking out ISG15 promotes autophagy and enhances cell survival after 5-FU chemotherapy
	24,452,380	2014	LC	A549	NA	Loss of autophagy reduces ISG15 expression through ROS mediated inhibition of S6K phosphorylation
Immune evasion	31,709,456	2019	PDAC	Panc02	Female C57BL/6 mice	ISG15 promotes PDAC immune escape by upregulating PD-L1
	37,217,923	2023	LUAD	A549, H1299, PC-9, and LLC	Female C57BL/6 mice	ISG15 promotes the degradation of glycosylated PD-L1 via K48-linked ubiquitination, reducing PD-L1 stability and enhancing antitumor immunity
	31,974,171	2020	BRCA	293 T	MMTV-PyMT mice	ISGylation enhances CD8 + cytotoxic T cells through the regulation of STAT1/STAT2 aggregation, promoting the production of CXCL9/10 chemokine
	38,926,796	2024	HNSCC	SCC25, FaDu, HT-29, BxPC-3, AsPC-1, U937, and MDA-MB-415	BALB/c nude mice	ISG15 promotes RAGE receptor activation through STING pathway and enhances tumor invasion and metastasis
	38,312,842	2024	Melanoma	B16-F10 and BPR20	Female C57BL/6 mice	ISG15 is significantly induced by STING agonist ADU-S100 and acts as an immunomodulator to limit its antitumor effect
	38,781,019	2024	ECA	OE33, HEK293, HeLa, and Jurkat	NA	ISG15 interacts with GRAIL1, a ubiquitin ligase, facilitating CD3 degradation and leading to immunosuppression in ECA
	33,424,843	2020	NPC	C666-1, HK1	NA	NPC cells secrete ISG15, which interacts with LFA-1 on macrophages, activating the SRC kinase-CCL18 axis, leading to M2-like macrophage polarization
	38,939,897	2024	OC	IOSOE385/386, OVCAR-3/4, HGSOC, TR127/182, and POCCs	Nude mice	ISG15 is highly expressed in ovarian-cancer-derived EVs and regulates EV secretion, tumor growth, and metastasis
Metabolic reprogram	38,982,116	2024	COAD and lymphoma	MC38, CT26, A20, HT29, and 293 T	C57BL/6 and BALB/c mice	IFN-I signaling enhances OXPHOS and ATP production in tumor cells in an ISG15-dependent manner, thereby promoting dendritic cell activation and antitumor immunity
	34,556,814	2021	BRCA	D3H2LN, Cos1, and HEK293T	NA	ISGylation enhances EGFR recycling, sustaining prolonged Akt signaling, leading to increased tumor cell proliferation and invasion
	33,380,466	2021	LC	393 T/P, A549, HOP62, H226, ED1, and 344SQ	FVB-USP18^−/−^ mice	USP18 promotes UCP1 expression and increases the oxidation rate of fatty acids:
	28,242,811	2017	LC	ED1, 344P, 393P, LKR13, H522, H2122, HOP62, and C10	Kras^LA2/+^/USP18^−/−^ mice	USP18 is upregulated in KRAS-mutant lung cancer and stabilizes KRAS protein
	29,367,604	2018	LC	A549 and HEK293T	Male athymic nude mice	ISGylation enhances the E3 ubiquitin ligase activity of CHIP and promotes c-Myc degradation
	30,765,861	2019	OSCC	HOK, CGHNC9, OC3, OEC-MI, LN1-1, TW2.6, SAS, and HSC3	Male BALB/cAnN mice	ISG15 promotes tumor cell migration by binding to Rac1 and is associated with poor prognosis
Cancer stem cell	35,506,701	2022	Glioma	U87, U251, and HEK293T	Nude mice	ISGylation of Oct4 protein increases the stem cell properties of glioma
	30,771,383	2019	PDAC	BxPC3, SW1990, and PANC-1	NA	BAG3 deletion reduces CSC-like properties in PDAC by suppressing ISG15 translation
	32,472,071	2020	PDAC	HPDE	Female NU-Foxn1^nu^ nude mice	Loss of ISG15 impairs mitophagy, leading to mitochondrial dysfunction and loss of PaCSC properties
	38,626,369	2024	BRCA	BT-549, Hs578T, MDA-MB-231, and MCF-7	Female nude mice	ISG15 promotes HNRNPA2B1-mediated BCSC maintenance through ISGylation and enhances dryness-related m6A mRNA nuclear output
	25,368,022	2014	PDAC	primary PDAC cultures	ISG15^+/+^ and ISG15^−/−^ mice	TAMs enhance CSC stemness by secreting ISG15 and promote the formation of M2 immune microenvironment
	37,501,099	2023	ATC	NT, NTHY, BCPAP, 8505C, and KHM5M	Nude mice and zebrafish model	ISG15 modifies KPNA2 through ISGylation to enhance ATC CSCs maintenance
	33,797,839	2021	OC	SKOV3 and A2780,	BALB/c-nu/nu mice	SG15 influences CSC-related properties and drug sensitivity through KLF12-mediated transcriptional inhibition
DNA damage response	38,976,080	2024	BLCA	J82, 5637, UM-UC-3, T24, SV-HUC-1, and HEK293T	Female BALB/c nude mice	PPP2R2B facilitates nuclear translocation of ISG15 via IPO5, inhibiting DNA repair and activating the STING pathway
	38,443,596	2024	NSCLC	HEK293T/FT, C-33A, HeLa, A549, MCF7, Huh7, H23, T47D, and HepG2	BALB/c nude mice	ISGylation of SIRT1 enhances its deacetylase activity, leading to increased DNA damage repair and drug resistance
	33,214,684	2021	CML and CRC	HAP1, PBMC, and HCT116	NA	USP18 deletion promotes DNA-induced interferon signaling and enhances the sensitivity of tumor cells to radiation therapy
	32,597,933	2020	OS, BRCA, CC, and GBM	U2OS, MCF7, HeLa, T98G, and M059K	NA	ISG15 localizes at DNA replication forks, interacting with PCNA and nascent DNA, regulating DNA synthesis
	39,456,585	2024	CRC	DLD-1, SW480, RKO, FET, and MC38	C57BL/6 mice	ISG15 enhances irinotecan resistance through NF-κB-dependent inflammation, CSC maintenance, and PD-L1-mediated immune escape
	31,926,942	2020	OC	A2780 and SKOV3	NA	ISGylation of hnRNPA2B1 inhibits ABCC2 translation, leading to increased cisplatin sensitivity in resistant cancer cells

5-FU, 5-fluorouracil; AML, acute myeloid leukemia; ATC, anaplastic thyroid cancer; BLCA, bladder cancer; BRCA, breast cancer; CC, cervical cancer; CML, chronic myeloid leukemia; COAD, colon adenocarcinoma; CRC, colorectal cancer; CSC, cancer stem cell; ECA, esophageal cancer; EGFR, epidermal growth factor receptor; EMT, epithelial–mesenchymal transition; EVs, extracellular vesicles; FS, fibrosarcoma; GBM, glioblastoma; HCC, hepatocellular carcinoma; HNSCC, head and neck squamous cell cancer; LC, lung cancer; LUAD, lung adenocarcinoma; melanoma, melanoma; MM, myeloma; NPC, nasopharyngeal carcinoma; NSCLC, non-small cell lung cancer; OC, ovarian cancer; OS, osteosarcoma; OSCC, oral squamous cell carcinoma; OXPHOS, oxidative phosphorylation; PaCSC, pancreatic cancer stem cell; PCNA, proliferating cell nuclear antigen; PDAC, pancreatic ductal adenocarcinoma; PD-L1, programmed death-ligand 1; ROS, reactive oxygen species; STING, stimulator of interferon genes; TAMs, tumor-associated macrophages

## ISGylation and autophagy regulation

Autophagy is a highly conserved intracellular degradation mechanism. In tumors, autophagy limits genomic instability on the one hand and supports tumor cell survival under conditions such as hypoxia and nutrient deprivation on the other hand [36, 37]. Autophagy consists of several stages: initiation, phagophore nucleation, autophagosome maturation, and fusion with lysosomes. It is coordinated by core proteins involved in autophagy, such as BECN1, ATG5, ATG7, and LC3 [38]. Conjugated ISG15 directly regulates autophagy by regulating the levels of key autophagy-related proteins. In breast cancer (BRCA) cells characterized by overactive Ras signaling, ISG15 promotes malignant transformation by preventing its lysosomal degradation through covalent modification mediated stabilization of Ki-Ras [39]. Similarly, under gemcitabine-induced chemotherapy stress, pancreatic ductal adenocarcinoma (PDAC) cells show increased ISGylation of the autophagy protein ATG7, thereby enhancing autophagic flow and treatment resistance [40]. It has also been reported that the loss of autophagy extension proteins ATG5 or ATG7 in tumor cells leads to the accumulation of double-stranded DNA (dsDNA) in the cytoplasm, which activates stimulator of interferon genes (STING) signaling pathway and increases the expression level of ISG15, thereby promoting the acquisition of tumor-related phenotypes, such as invasion and proliferation [41]. Notably, ISG15 under these conditions is mainly secreted in the free form, rather than in the classical covalent binding pathway. In addition, knockdown of ISG15 or UBE2L6 in chemotherapy-sensitive esophageal cancer (ECA) cells enhances autophagy, and only ISG15 knockout enhances both autophagy and chemotherapy-induced cell survival [42]. In non-small cell lung cancer (NSCLC), IFN-I drives HERC5-dependent ISGylation of the E3 ligase TRIM21, which enhances its enzymatic activity. The activation of TRIM21 causes the protein p62 to undergo ubiquitin modification, which hampers the oligomerization of p62 and stops the formation of the autophagosome, hence stopping autophagic degradation [43]. This contrasts with impaired autophagy (e.g., loss of ATG5 or Beclin-1), in which the accumulation of reactive oxygen species (ROS) leads to decreased S6 kinase activity and inhibits the transcription and protein expression of ISG15, rendering tumor cells that are resistant to oncolytic viruses susceptible [44]. This context-dependent duality illustrates the complexity of ISG15-mediated regulation of autophagy. The above related core literature information is presented in Table 1, and Fig. 1B shows the proposed mechanisms.

## ISGylation and immune evasion

Tumor cells survive and metastasize by immune escape mechanisms, thereby evading recognition by the host immune system. It mainly remodels TME by downregulating antigen presentation, secreting immunosuppressive cytokines, and upregulating immune checkpoint molecules [45, 46]. ISG15 and ISGylation affect the response of tumor cells to the immune system by affecting antigen presentation and immune cell activity. First, ISG15 is involved in the regulation of PD-L1 expression. Burks et al. [47] found that ISG15 knockdown not only suppresses KRAS-driven malignant phenotype, but also significantly downregulates PD-L1 expression and enhances tumor infiltration of CD8^+^ T cells. Free ISG15 can act as a cytokine to upregulate PD-L1 by activating IRF-1 signaling. ISG15 knockdown can reduce the tumor invasion of Foxp3^+^ regulatory T cells and produce a synergistic effect with anti-PD-1 antibody treatment. In lung adenocarcinoma (LUAD), Qu et al. [48] found that ISG15 reduces the expression level of PD-L1 by modifying glycosylated PD-L1 to promote its K48-linked ubiquitination. Notably, despite low ISGylation activity in mice, overexpression of ISG15 significantly enhances the therapeutic effect of anti-PD-L1 in a mouse model of LC, suggesting that even limited ISGylation can effectively mediate PD-L1 degradation when ISG15 is locally expressed. This study provides evidence for the involvement of ISG15 in PD-L1 regulation, while underlining the need for further mechanistic validation in humanized settings.

In terms of regulating innate immunity, the regulation of ISG15 in the cyclic guanosine monophosphate–adenosine monophosphate synthase (cGAS)-STING pathway shows a dual role. In melanoma, the STING agonist ADU-S100 can significantly induce the expression of ISG15, while neutralizing ISG15 can enhance the maturation of dendritic cells and the activation of CD8^+^ T cells [49]. In contrast, Li et al. [50] found that in head and neck squamous cell carcinoma (HNSCC), cytoplasmic dsDNA released by necroptosis activated the cGAS-STING pathway, thereby upregulating ISG15 expression. At this point, ISG15 promotes the recruitment of immune cells by binding to the RAGE receptor [50]. The discrepancy may reflect ISG15 engaging distinct receptors under different immune contexts, thereby triggering divergent effects. As Chen et al. [51] found in nasopharyngeal carcinoma, ISG15 interacts with its receptor LFA-1 to polarize tumor-associated macrophages (TAMs) to the M2 phenotype, inhibit the effector function of CD8 + T cells, and thus engender an immunosuppressive microenvironment.

ISG15 has a significant tumor type-specific pattern of immune regulation. In cervical squamous cell carcinoma, Zhao et al. [52] observed that the downregulation of ISG15 expression is associated with enhanced tumor invasiveness. McEwen et al. [53] reported that ISG15 impairs T cell function in ECA by specifically promoting the degradation of CD3-ε/γ/δ peptides. In contrast, in BRCA, ISG15 enhances STAT1/2 activity through UBA7-mediated ISGylation, thereby increasing the expression of chemokines such as CXCL9/10/11 [54]. In addition, Dorayappan et al. [55] found that ISG15 promotes the formation of multivesicular body and inhibits their fusion with lysosomes, thereby enhancing the secretion of extracellular vesicles (EVs) in ovarian cancer (OC). ISGylation can also stabilize STAT3 protein and promote its release into the tumor microenvironment with EVs, which may induce immune tolerance. Taken together, ISG15 is a key hub connecting innate immunity, adaptive immunity, and immune checkpoint regulation. The above related core literature information is presented in Table 1, and Fig. 1C shows the proposed mechanisms.

## ISGylation and tumor metabolic reprogramming

Metabolic reprogramming is a hallmark of tumorigenesis and progression. The metabolism of tumor cells is altered to meet the demands of energy and biosynthetic precursors for rapid proliferation [56, 57]. This not only supports tumor growth, but also contributes to remodeling of the TME and drug resistance, contributing to malignant progression [58]. In BRCA, ISG15 modifies GDI2 through ISGylation to reduce its binding ability to Rab proteins (Rab5/11), which mainly regulates epidermal growth factor receptor (EGFR) recycling or retrograde trafficking to the Golgi. ISGylation reduces the interaction between GDI2 and Rab proteins, and more Rab proteins exist in the free active state to promote EGFR recycling from the cell membrane more rapidly, ensuring the persistent presence of EGFR on the cell membrane and continuously activating the downstream PI3K-Akt signaling pathway [59]. A similar ISGylation-dependent mechanism is also observed in LC, where KRAS signaling critically depends on its stable localization to the plasma membrane. When KRAS is modified by ISG15, it leads to mistranslocation of KRAS from the cell membrane to the inner membrane system. USP18 removes ISG15 from KRAS to prevent its mislocalization and ensure the continuous activation of downstream PI3K-Akt and MAPK signaling [60]. The study by Liu et al. [60] further points to a dual mechanism by which USP18 promotes LC growth. First, USP18 stabilizes ATGL by removing the ISG15 conjugation, thereby enhancing lipolysis. Second, USP18 promotes mitochondrial fatty acid oxidation by increasing the expression of UCP1, a mitochondrial inner membrane protein that facilitates thermogenic respiration and lipid metabolism. Both mechanisms work together to support tumor cell proliferation. ISG15 also coordinates immune–metabolic interactions in the tumor microenvironment. Zhou et al. [61] found that IFN-I directly enhanced mitochondrial oxidative phosphorylation (OXPHOS) in tumor cells by inducing the expression of ISG15. The metabolic shift satisfies the energy demand of tumor cells on the one hand and induces the release of extracellular ATP from autophagy on the other hand. Extracellular ATP then activates dendritic cells via P2X7 receptors to enhance antitumor responses, with a synergistic effect with CD47-SIRPα blockade immunotherapy. In addition, Yoo et al. [62] found that the E3 ubiquitin ligase CHIP enhances its enzymatic activity through ISGylation, which increases its degradative capability toward the oncogenic transcription factor c-Myc. Likewise, free ISG15 interactions contribute to the metabolic fitness of cancer cells. Chen et al. [63] found that ISG15 can directly bind to Rac1-GDP and promote Rac1 activation and membrane protrusion formation in oral squamous cell carcinoma, which in turn promotes actin remodeling and cell motility. This migration phenotype is closely related to cytoskeleton remodeling and ATP local energy supply. Taken together, ISG15 acts as a dynamic metabolic regulator and participates in multiple signaling networks and metabolic pathways in tumors. The above related core literature information is presented in Table 1, and Fig. 1D shows the proposed mechanisms.

## ISGylation in cancer stem cell maintenance

CSCs are regarded as critical contributors to tumor recurrence and therapeutic resistance, primarily due to their self-renewal capacity and inherent drug resistance [64, 65]. ISG15 regulates pancreatic cancer stem cell (PaCSC) properties through different forms of action. Alcala et al. [66] found that the maintenance of stemness of PaCSCs is dependent on intracellular covalent ISGylation, and loss of ISG15 results in significant mitochondrial dysfunction in PaCSCs, which is due to Parkin degradation. Phenotypic analysis shows that ISG15 deficiency enhances the sensitivity of PaCSCs to metabolic inhibitors and ultimately inhibits PaCSCs self-renewal and tumorigenesis, suggesting the potential of ISG15 as a target for intervention. Li et al. [67] found that knockdown of either BAG3 or ISG15 significantly inhibits CSC phenotypes such as colony formation, spheroid formation, and CD44 expression in PDAC cells. Importantly, there is no significant difference in the ability of ISGylation-deficient mutants and wild type ISG15 to restore CSC properties in the overexpression model, suggesting that the free ISG15 can independently maintain CSC properties. In addition to intracellular effects, TAMs in the TME of PDAC abundantly secrete free ISG15 when stimulated by IFN-I secreted by PaCSCs. Furthermore, ISG15 significantly increases the self-renewal ability of PaCSCs and activates the ERK pathway in a paracrine manner [68]. In contrast to PDAC, the free ISG15 in OC has a significant inhibitory function on CSC identity. Zhang et al. [69] found that KLF12, as a transcriptional inhibitor, indirectly enhances CSC identity by inhibiting ISG15 transcription in OC. PCAT6, which is expressed under hypoxia, could serve as a backbone molecule to mediate covalent modification of hnRNPA2B1 by ISG15. Subsequently, hnRNPA2B1 protein binds to m6A-modified CSC-related mRNAs to promote self-renewal of BRCA stem cells [70]. In glioma and anaplastic thyroid carcinoma (ATC), ISG15 inhibits the ubiquitination and degradation of key molecules by ISGylation to activate the expression of downstream stemness genes and enhance tumor stemness characteristics and drug resistance [71, 72]. Many studies have shown that free ISG15 enhances the self-renewal, invasion, and migration abilities of CSCs mainly through extracellular signal transduction pathways. Further, covalent ISGylation regulates stem-cell-specific metabolism and the function of key transcription factors through stability regulation. The above related core literature information is presented in Table 1, and Fig. 1D shows the proposed mechanisms.

## ISGylation in DNA damage repair and drug resistance

DDR is essential for maintaining genome stability, especially in the face of continuous stimulation by endogenous factors such as replication stress, R-loop accumulation, and various exogenous genotoxic damage [73, 74]. High levels of free ISG15 are found to anchor near replication forks and interact with proliferating cell nuclear antigen (PCNA) and DNA helicase RECQ1 to accelerate forward extension. Although the rate of replication is faster, it is also more likely to produce double-strand breaks and chromosome aberrations. This also makes tumor cells with high ISG15 expression more susceptible to cell death when exposed to DNA-damaging drugs [75]. However, it has also been reported that ISG15 is upregulated by irinotecan treatment in colorectal cancer (CRC) cells, accompanied by the co-elevation of multiple resistance factors (e.g., osteopontin, survivin) [76]. Several studies have highlighted the inhibitory effect of ISG15 on DDR. Huang et al. [77] found that PPP2R2B inhibits DNA repair and enhances cisplatin sensitivity by promoting the conjugation of ISG15 to nuclear import protein IPO5 in bladder cancer (BLCA), and the accumulated DNA damage activates the STING pathway, initiating a positive feedback loop that enhances ISG15 transcription. This suggests that ISG15 is not only a product of interferon induction, but is itself involved in the regulation of DDR. In addition to free ISG15, ISGylation is involved in the regulation of DDR in cancer cells to affect drug response. Kang et al. [78] found that ISG15 conjugation to SIRT1 disrupts its binding to the repressor DBC1, thereby enhancing SIRT1 activity in LC cells. DNA damage is then inhibited by further deacetylation of multiple downstream proteins. In addition, Wang et al. [79] found that ISG15 interferes with its binding to ABCC2 mRNA by covalently modifying hnRNPA2B1 in OC. As a transporter, ABCC2 will pump the drug out of the cell in the cisplatin environment to reduce the intracellular drug concentration. Therefore, the increased expression of ISG15 can effectively reduce the expression of ABCC2 and improve the sensitivity of tumor cells to cisplatin. In terms of radiotherapy, Pinto-Fernandez et al. [80] found that in the absence of USP18, ADAR is modified by ISG15 with decreased enzyme activity and is unable to deaminate dsRNA. Intracellular dsRNA accumulation can activate PKR and enhance DNA damage response, enhancing the sensitivity of tumor cells to radiotherapy. Furthermore, the regulation of ISG15 modification is not limited to DDR, but also enhances DNA damage by interfering with RNA homeostasis and immune sensing pathways. The above related core literature information is presented in Table 1, and Fig. 1E shows the proposed mechanisms.

## Perspective

Given the steady increase in the global burden of cancer, it is of great significance to interpret new regulatory mechanisms such as ISGylation [81]. Despite the growing body of evidence supporting the multifaceted role of ISG15 and ISGylation in tumors, the regulatory network is still not fully understood. A key point lies in the fact that ISG15 exists in both free and conjugated forms. Free ISG15, especially when secreted outside the cell, acts as a cytokine-like molecule to regulate immune responses, whereas conjugated ISG15 mainly changes the stability, localization, and function of substrate proteins in the intracellular environment [82]. The two molecular states may play opposite or complementary roles depending on the cellular context. Moreover, unlike ubiquitination, which is highly conserved, ISG15 is highly divergent even in mammals [83]. For example, influenza B virus NS1B protein can only bind to ISG15 in humans and nonhuman primates and cannot antagonize ISGylation in mice [84]. Of note, there are clear species-specific differences in the mechanisms of ISGylation between humans and mice. In human cells, HERC5 plays a major role as an ISG15 E3 ligase, whereas mice lack the HERC5 homolog and instead utilize HERC6 for ISGylation [85]. This discrepancy presents a hurdle for preclinical studies, and the result is that murine models may not fully reflect the effects of ISG15 on human cancers. Therefore, the development of humanized models or alternative platforms is essential to bridge this gap.

ISG15 also functions as a potential biomarker. Overexpression of ISG15 has been frequently observed in various tumors [86]. In BRCA, ISG15 expression correlates with multiple clinicopathological features, including lymphovascular invasion and histological grade [87–89]. However, its clinical applicability faces several challenges. Firstly, ISG15 exhibits varying expression patterns and functions across different tumor types, necessitating further clarification of its role in specific tumor types and molecular subtypes to enhance its diagnostic precision. Secondly, the detection methods for ISG15 as a biomarker require optimization. Currently, most studies utilize immunohistochemistry and western blot for ISG15 expression analysis; however, the feasibility of these methods in clinical applications is limited. It may be beneficial to further investigate the potential of detecting ISG15 in blood or urine and evaluate whether it can serve as a biomarker for predicting immunotherapy response or disease progression. For instance, current findings indicate that ISG15 mRNA levels in the blood of systemic lupus erythematosus patients are significantly higher than those in healthy controls, and ISG15 expression is closely linked to disease activity [90]. ISG15 needs to be integrated with other known biomarkers in future research. Currently, PD-L1, tumor mutational burden, and microsatellite instability are widely used to predict the efficacy of immunotherapy. ISG15 may regulate the tumor microenvironment by modulating PD-L1 stability, thereby influencing the effectiveness of immunotherapy. Therefore, future clinical studies should explore the correlation between ISG15 and existing immunotherapy biomarkers. Additionally, ISG15 could serve as a potential target for cancer vaccines, such as the ISG15-based Listeria vaccine, which has demonstrated good immunogenicity in CRC immunotherapy [91].

From a therapeutic perspective, the reversibility of ISGylation opens the possibility to manipulate this pathway. In drug development, inhibitors targeting the enzyme UBA1, which is involved in ubiquitin binding, have entered clinical trials. Additionally, small molecule inhibitors of the ISG15 cross-reactive deubiquitinase USP24 have been discovered to suppress drug resistance in chemotherapy [92, 93]. A recent preprint further demonstrates that USP24 functions as an ISG15 cross-reactive deubiquitinating enzyme [94]. Although detailed reports on UBA7-specific inhibitors targeting ISGylation have not yet emerged, recent structural studies have unveiled the architecture of UBA7 and its interactions with UBE2L6 and ISG15 adenylate [95]. Beyond directly targeting ISG15 and its associated proteins, it may also be possible to intervene by regulating downstream proteins such as Bcl-2 and STAT2. Furthermore, proteolysis-targeting chimeras (PROTACs) may represent a promising intervention strategy [96]. PROTACs guide the target protein to the E3 ligase by forming a ternary complex, ultimately triggering its degradation. Currently, ARV-110, the first PROTAC designed to degrade the androgen receptor, is in clinical trials for metastatic prostate cancer. Although PROTACs with ISG15 specificity have not yet been designed, the clinical advancement of ARV-110 indicates the therapeutic potential of PROTAC technology and provides a framework for drug development in the ISG15. It is anticipated that, with the advancement of artificial intelligence drug screening technology, the development of highly specific ISG15-targeting drugs will be feasible in the future. Given the complexity and heterogeneity of tumors, an integrated multi-omics approach is essential to uncover the regulatory networks that drive cancer progression [97]. Future efforts should focus on deconstructing the ISGylation landscape at single-cell resolution, mapping the substrate specific network, and identifying reliable biomarkers of ISG15 activity. Ultimately, a deeper understanding of the dynamic mechanisms of ISGylation will help translate this unique ubiquitin-like modifier into a viable target for cancer therapy.

## Supplementary Information

Additional file 1. Figure S1. Overview of ISGylation and its roles in cancer-related phenotypes.

## Data Availability

Not applicable. No new data were generated or analyzed in this study.

## Abbreviations

ATC: Anaplastic thyroid cancer
BRCA: Breast cancer
BLCA: Bladder cancer
CRC: Colorectal cancer
COAD: Colon adenocarcinoma
CSCs: Cancer stem cells
DDR: DNA damage response
dsDNA: Double-stranded DNA
ECA: Esophageal cancer
EGFR: Epidermal growth factor receptor
EMT: Epithelial–mesenchymal transition
EVs: Extracellular vesicles
HCC: Hepatocellular carcinoma
HNSCC: Head and neck squamous cell cancer
ICIs: Immune checkpoint inhibitors
IFN-I: Type I interferon
ISG15: Interferon-stimulated gene 15
ISGs: Interferon-stimulated genes
LC: Lung cancer
LUAD: Lung adenocarcinoma
NSCLC: Non-small cell lung cancer
OC: Ovarian cancer
OXPHOS: Oxidative phosphorylation
PD-L1: Programmed death-ligand 1
PDAC: Pancreatic ductal adenocarcinoma
PROTACs: Proteolysis-targeting chimeras
PTM: Post-translational modification
PaCSCs: Pancreatic cancer stem cells
ROS: Reactive oxygen species
STING: Stimulator of interferon genes
TAMs: Tumor-associated macrophages
TME: Tumor microenvironment
