# Supplementary figures and images for: The ISGylation tapestry in cancer: weaving phenotypic plasticity through multidimensional regulatory looms
Source: Cell Mol Biol Lett. 2025 Nov 5;30:132. doi: 10.1186/s11658-025-00815-6 (PMC12590619; doi:10.1186/s11658-025-00815-6)

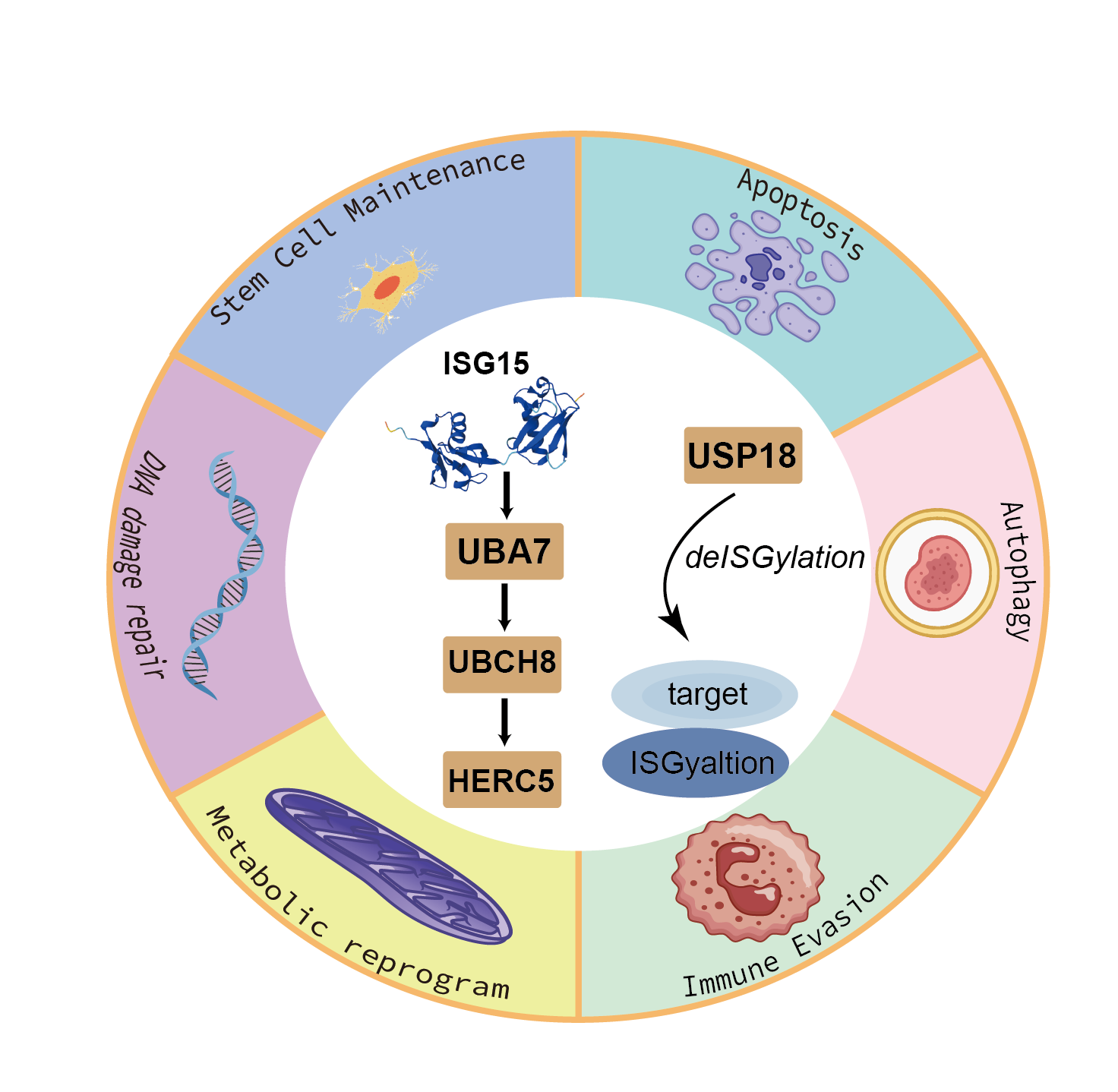

Supplement: Supplementary file 1 — Additional file 1. Figure S1. Overview of ISGylation and its roles in cancer-related phenotypes. [file 11658_2025_815_MOESM1_ESM.png]
